# Delay-Fluctuation-Resistant Underwater Acoustic Network Access Method Based on Deep Reinforcement Learning

**DOI:** 10.3390/s25216673

**Published:** 2025-11-01

**Authors:** Jinli Shi, Kun Tian, Jun Zhang

**Affiliations:** School of Electronic and Information Engineering, South China University of Technology, Guangzhou 510641, China; 202420111707@mail.scut.edu.cn (J.S.); a1067552189@163.com (K.T.)

**Keywords:** underwater acoustic sensor network, media access control, delay fluctuation, deep reinforcement learning

## Abstract

The slow propagation speed of acoustic waves in water leads to significant variations and random fluctuations in communication delays among underwater acoustic sensor network (UASN) nodes. Conventional deep reinforcement learning (DRL)-based underwater acoustic network access methods can adaptively adjust their parameters and improve network communication efficiency by effectively utilizing inter-node delay differences for concurrent communication. However, they still suffer from shortcomings such as not accounting for random delay fluctuations in underwater acoustic links and low learning efficiency. This paper proposes a DRL-based delay-fluctuation-resistant underwater acoustic network access method. First, delay fluctuations are integrated into the state model of deep reinforcement learning, enabling the model to adapt to delay fluctuations during learning. Then, a double deep Q-network (DDQN) is introduced, and its structure is optimized to enhance learning and decision-making in complex environments. Simulations demonstrate that the proposed method achieves an average improvement of 29.3% and 15.5% in convergence speed compared to the other two DRL-based methods under varying delay fluctuations. Furthermore, the proposed method significantly enhances the normalized throughput compared to conventional Time Division Multiple Access (TDMA) and DOTS protocols.

## 1. Introduction

The propagation speed of underwater acoustic signals ranges from approximately 1460 m/s to 1520 m/s, which is much lower than that of radio frequency signals ($3\times 10^{8}$ m/s). This dramatically reduced propagation speed introduces two critical challenges for underwater acoustic sensor networks (UASNs). First, the slow signal velocity creates significant end-to-end latency, with one-way delays often reaching hundreds of milliseconds even for moderate distances. Second, environmental factors including temperature gradients, salinity variations, and dynamic water currents cause temporal variations in propagation speed, leading to significant propagation delay fluctuation. These physical characteristics make the collision patterns in UASNs completely different from those on land and significantly affect the performance of the Medium Access Control (MAC) protocol in UASNs.

To address the propagation delay variations caused by the different distances between source nodes and target nodes, some MAC protocols for UASNs regard them as parallel opportunities in time. By strategically scheduling the transmission times of source nodes, these protocols enable collision-free concurrent transmission in time division multiple access (TDMA) [1,2,3,4] or handshake-based [5,6,7] schemes, or reduce the collision probability in random access protocols [8]. However, these protocols generally depend on accurate propagation delay estimation, and the dynamic underwater environments often undermine delay estimation accuracy and protocol reliability.

In recent years, DRL [9,10] has introduced a novel paradigm for enhancing communication protocols in UASNs. DRL algorithms allow network nodes to learn through environment interaction, thereby optimizing key functions such as routing, resource allocation, and dynamic spectrum access. This capability improves the adaptability and overall performance of the network. Currently, research on DRL-based MAC protocols for UASNs is still in the early stage. Only a limited number of such protocols have been proposed, which aim to enhance network throughput by efficiently utilizing the idle time slots resulting from propagation delay differences among nodes. In [11], a DRL-based MAC protocol for UASNs named DR-DLMA (Delayed Reward Deep Reinforcement Learning Multiple Access) was first introduced. It formalized the state, action, and reward structures within the UASN access process and integrated propagation delay into the DRL framework. In the DR-DLMA protocol, nodes apply deep reinforcement learning to select the optimal channel access strategy. This approach effectively utilizes the idle time slots caused by the propagation delay differences or unused slots from other nodes to improve data transmission efficiency. In [12], the AUMA (Adaptive Underwater Medium Access Control based on Deep Reinforcement Learning) protocol was proposed. Leveraging DRL techniques, it effectively utilizes the high propagation delay of UASNs to learn the optimal round-trip transmission delay and window size, and adjusts the congestion window mechanism to improve the overall throughput and channel utilization of high-mobility underwater acoustic communication networks. In [13], the issue of unfair channel allocation and energy-constrained nodes in heterogeneous hybrid optical–acoustic UASNs was addressed through a new DRL-based MAC protocol.

Although conventional DRL-based MAC protocols for UASNs account for propagation delay in reward acquisition, most do not consider the delay fluctuations during an agent’s transition from the current state to the next. Specifically, during state transition, an agent must wait for a duration equivalent to round-trip propagation delay to obtain state observations. Due to the low propagation speed of underwater acoustic signals, the actual delay may vary within this waiting period, thereby affecting the convergence speed and stability of the network. Additionally, the DRL networks employed in these protocols are relatively simple, which consequently results in insufficient learning efficiency. To address these problems, this paper proposes a DRL-based delay-fluctuation-resistant UASN access method. First, delay fluctuations are integrated into the state model of deep reinforcement learning, enabling the model to adapt to delay fluctuations during learning. Then, a double deep Q-network (DDQN) is introduced, and its structure is optimized to enhance learning and decision-making in complex environments.

The structure of this paper is organized as follows: Section 2 reviews the works related to MAC protocols for UASNs. Section 3 introduces the new delay-fluctuation-resistant DRL model; Section 4 describes the proposed delay-fluctuation-resistant UASN access method based on DDQN; Section 5 presents and discusses the simulation results; Finally, Section 6 concludes the paper.

## 2. Related Work

### 2.1. Conventional MAC Protocols for UASNs

The MAC protocol, which manages access to the shared broadcast channel among nodes in a network, is a key technology for UASNs. These MAC protocols are generally classified into three categories: non-contention-based, contention-based, and hybrid.

Non-contention-based MAC protocols, such as TDMA [14], frequency division multiple access (FDMA) [15], and code division multiple access (CDMA) [16], divide spectrum resources into subchannels by time, frequency, or code and allocate them statically to nodes. These protocols typically manage uplink and downlink communications through centralized resource allocation. This avoids collisions and suits heavy, continuous, and balanced traffic, but may waste resources under light or bursty loads.

Contention-based protocols allow nodes to dynamically compete for channel access, making them suitable for light, bursty, or unbalanced traffic. These protocols primarily focus on uplink access cases where multiple nodes compete to send data to a common receiver. However, since channel competition may lead to collisions which significantly reduce transmission efficiency, how to mitigate or eliminate collisions remains a key research focus in contention-based protocols. Contention-based protocols are further divided into random access protocols (e.g., ALOHA [17] and CSMA (Carrier Sense Multiple Access) [18]), where nodes transmit without coordination and risk collisions, and handshake-based protocols (e.g., MACA (Multiple Access with Collision Avoidance) [19] and FAMA (Floor Acquisition Multiple Access) [20]), which use RTS/CTS exchanges to avoid collisions.

Hybrid MAC protocols are mainly designed for uplink access in UASNs. They combine multiple mechanisms (e.g., non-contention with contention, or different subtypes within each category [21]) and adaptively switch between them to leverage their respective advantages, aiming for improved overall performance in varying network conditions.

### 2.2. UASN MAC Protocols Based on Reinforcement Learning

The advancement of intelligent algorithms, such as Reinforcement Learning (RL) and DRL, has opened new pathways for enhancing communication protocols. These sophisticated algorithms enable nodes to autonomously optimize tasks like routing, resource allocation, and dynamic spectrum access through interaction with the environment. However, research on intelligent MAC protocols for UASNs remains in its early stages, often involving adaptations of protocols originally designed for terrestrial wireless networks. RL-based intelligent MAC protocols learn optimal behaviors through reward feedback, making them suitable for simple environments. Yet, their effectiveness is limited by the size of the state space. In contrast, DRL-based protocols integrate deep learning to handle more complex environments and broader state spaces, albeit at the cost of increased computational resources and data requirements.

In the study of RL-based intelligent MAC protocols for UASNs, several innovative approaches have been proposed. For instance, a new method that combines reinforcement learning with Slotted-CSMA was proposed in [22]. This protocol focuses on uplink access cases where sensor nodes transmit data toward surface stations. It divides the underwater acoustic channel into multiple subchannels and employs Q-learning to help nodes select both the optimal relay node and the most suitable subchannel for data transmission. Another study introduced RL-MAC (Reinforcement Learning-based MAC) [23], which is designed to address challenges such as propagation delay, error probability, node mobility, and low data rates in underwater multimedia sensor networks. By applying Q-learning, this protocol optimizes the contention mechanism during the initial phase of multimedia transmission, thereby improving both transmission efficiency and overall network performance. Additionally, the ALOHA-QUPAF (Packet flow ALOHA with Q-learning) protocol [24] was developed as a packet-flow-based reinforcement learning MAC protocol. To address linear multi-hop uplink cases, it uses an improved two-phase Q-learning process to extract implicit reward signals, further optimizing data transmission in underwater acoustic sensor networks. The DR-ALOHA-Q (Delayed-reward ALOHA-Q) protocol proposed in [17] is a MAC protocol based on reinforcement learning within the ALOHA framework. This protocol supports asynchronous network operations and leverages long propagation delays to enhance network throughput.

Turning to DRL-based intelligent MAC protocols for UASNs, researchers have developed more advanced solutions capable of handling greater complexity. The research and design of these protocols primarily focus on uplink access cases, as the uplink concentrates the most challenging network issues (e.g., multi-node competition, energy efficiency optimization). Ye et al. pioneered the DR-DLMA protocol [11], which aims to maximize network throughput. Using deep reinforcement learning, DR-DLMA enables nodes to collaborate effectively within the network. It intelligently selects optimal channel access strategies, making efficient use of idle slots or unused slots caused by long propagation delays or other nodes’ activities, thereby optimizing overall network performance. Similarly, Geng et al. proposed the DL-MAC (Deep-reinforcement Learning-based Medium Access Control) protocol [25], which leverages deep reinforcement learning to exploit long propagation delays in underwater acoustic communication. This protocol adopts both synchronous and asynchronous transmission modes to enhance system throughput. Notably, its asynchronous mode adapts to spatiotemporal variations in UASNs by adjusting the start time of transmission slots, further refining network performance. Moreover, Liu et al. investigated efficient channel bandwidth utilization in heterogeneous hybrid optical–acoustic underwater sensor networks and proposed a novel MAC protocol based on deep reinforcement learning [26], demonstrating the continued evolution and application of DRL techniques in challenging underwater environments.

## 3. Delay-Fluctuation-Resistant DRL Model

To simplify the system design and focus on the performance analysis of the MAC layer mechanism, we assume that the physical layer can provide a stable bitstream service to the upper layer (MAC layer). Therefore, when constructing the DRL model and designing the access strategy, we do not consider specific underwater acoustic channel models (e.g., multipath, attenuation), but concentrate on MAC layer influencing factors such as propagation delay and delay fluctuations caused by variations in propagation speed.

In the DR-DLMA model, the system state $s_{t+1}$ is determined by the action $a_{t-2D}$ and the observation $o_{t}$, where D denotes the one-way propagation delay between the agent node and the master node. Specifically, $a_{t-2D}$ represents the action taken at timeslot t−2D, and $o_{t}$ represents the observation obtained after executing action $a_{t-2D}$. However, due to the slow propagation speed of underwater acoustic signals, the value of D is subject to random fluctuations during the agent’s transition from the current state to the next. These variations can be caused by environmental and dynamic factors such as water currents, node mobility, and changes in sound speed. DR-DLMA does not account for the impact of such delay fluctuations, which limits its ability to adapt to dynamic underwater delay conditions and affects the convergence speed and stability of the model.

In this paper, we incorporate a propagation delay fluctuation feature, ∆D, into the DRL framework by redefining the system state. The new state is a tuple composed of the delay fluctuation ∆D, the historical action $a_{t-2D}$, and the corresponding observation $o_{t}$, denoted as:

(1) $$w_{t+1}≜(∆D,a_{t-2D},o_{t})$$

The state $s_{t+1}$ of the agent node in time slot t+1 is defined as follows:

(2) $$s_{t+1}≜(w_{t-M+2},w_{t+1})$$

where M represents the historical length of the state.

The reinforcement learning elements [11] in this paper are defined as follows:

Agent: A network node that runs the DRL protocol.

Action: Each node can choose between two types of actions: transmitting and waiting. Therefore, the action set is defined as U={Transmit,wait}. In each time slot t, the node running the DRL protocol selects an action $a_{t}\in U$.

State: The system state is defined using Equations (1) and (2), incorporating the propagation delay fluctuations, the historical actions, and the corresponding observations.

Reward: At time slot t and corresponding state $s_{t}$, the node executes action $a_{t}$. Then, at time slot t+2D+1, it evaluates the performance of action $a_{t}$ and obtains a reward $r_{t+2D+1}$. The node transitions to a new state $s_{t+2D+1}$ simultaneously. The reward $r_{t+2D+1}$ is determined based on the reception result $o_{t+2D+1}$ of the master node, defined as follows:

(3) $$r_{t+2D+1}=\left\{\begin{array}{c} 1,o_{t+2D+1}>0\\ 0,o_{t+2D+1}\leq 0 \end{array}\right.$$

when $o_{t+2D+1}>0$, it indicates that the node has successfully transmitted, and the corresponding reward $r_{t+2D+1}$ is 1; when $o_{t+2D+1}\leq 0$, it indicates that the node was idle or a collision occurred in that time slot, so the reward $r_{t+2D+1}$ is 0.

## 4. Delay-Fluctuation-Resistant UASN Access Method Based on DDQN

In the DR-DLMA, the Deep Q-Network (DQN) algorithm [27,28] is employed. The DQN architecture consists of fully connected layers with residual connections. However, this neural network structure presents several limitations in practice. First, during *Q*-value updates, DQN selects the action corresponding to the maximum expected reward in the next state. This approach may result in a systematic overestimation of future rewards, as it consistently prioritizes the highest estimated values without accounting for potential uncertainties or environmental dynamics. Such overestimation leads to inaccurate policy evaluation during training, ultimately affecting the quality and performance of the learned policy. Second, the network structure in DQN is relatively simple and lacks an explicit mechanism for separating state values and action advantages of reinforcement learning, which may lead to low learning efficiency. Third, during updates, the neural network modifies the *Q*-value only for the executed action, and does not affect the *Q*-values of other actions. This update strategy limits the network’s ability to learn across the entire action space. To address these issues, this paper introduces the DDQN algorithm into the proposed delay-fluctuation-resistant DRL model. We also optimize the deep neural network structure in DDQN by separately modeling state values and action advantages, thereby enhancing its overall learning efficiency and policy performance.

### 4.1. Delay-Fluctuation-Resistant DRL Model Based on DDQN

Compared with the DQN, DDQN employs two networks with the same structure but different parameters to alleviate the issue of *Q*-value overestimation [29,30]. One network, called the main network, is responsible for selecting actions, while the other network, called the target network, is used to evaluate the value of these actions. DDQN utilizes the main network to select the optimal action $a^{*}$ for the next state as:

(4) $$a^{*}=arg\underset{a^{\prime }}{\max}Q(s_{t+1},a^{\prime };\theta )$$

where θ denotes the parameters of the main network, and $a^{\prime }$ denotes the action selected in the state $s_{t+1}$. Then, the target network is used to calculate the target *Q*-value for this chosen action as:

(5) $$y=r_{t+1}+\gamma Q(s_{t+1},a^{*};\theta^{-})$$

where $r_{t+1}$ denotes the reward obtained at time slot t+1, γ denotes the discount factor, and $\theta^{-}$ denotes the parameters of the target network.

To adapt DDQN to the proposed model, we modify its experience replay mechanism by adopting the delayed experience replay mechanism proposed in [11]. During the training process, a predefined number of real experiences $e_{t}$ are randomly sampled from the experience buffer to update the parameters of the main network. $e_{t}$ is defined as:

(6) $$e_{t}=(s_{t},a_{t},r_{t+2D+1},s_{t+2D+1})$$

where $e_{t}$ is constructed by combining the state $s_{t}$ and the action $a_{t}$ of the sequential experience ${\hat{e}}_{t}=(s_{t},a_{t},r_{t+1},s_{t+1})$ with the reward $r_{t+2D+1}$ and next state $s_{t+2D+1}$ of the sequential experience ${\hat{e}}_{t+2D}=(s_{t+2D},a_{t+2D},r_{t+2D+1},s_{t+2D+1})$. The state $s_{t}$ is given by (1) and (2). To account for the round-trip propagation delay 2D between the agent node and the master node, the reward $r_{t+1}$ and the state $s_{t+1}$ in (4) and (5) are replaced by $r_{t+2D+1}$ and $s_{t+2D+1}$ respectively. (4) and (5) are adjusted as follows:

(7) $$a^{*}=arg\underset{a^{\prime }}{\max}Q(s_{t+2D+1},a^{\prime };\theta )$$

(8) $$y=r_{t+2D+1}+\gamma Q(s_{t+2D+1},a^{*};\theta^{-})$$

### 4.2. Optimization of Neural Network Structure

**Figure 1:**
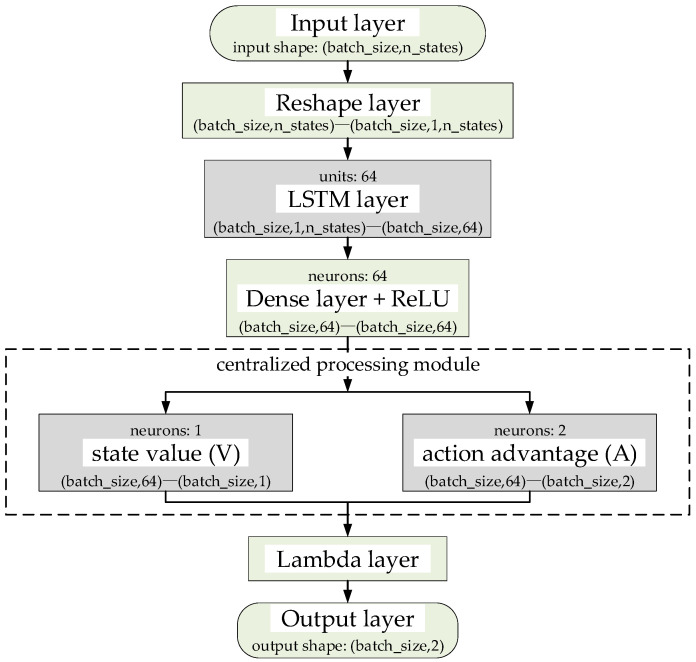
The improved neural network structure.

To address the issues of a simplistic neural network architecture and low learning efficiency in DR-DLMA, this paper proposes an improved network structure, as illustrated in Figure 1. Compared with DR-DLMA, a long short-term memory (LSTM) and a centralized processing module are added.

The proposed neural network takes a state vector of shape (batch_size,n_states) as input, where n_states denotes the state history length. This vector is reshaped into a 3D tensor of (batch_size,1,n_states) to fit the LSTM input format. The LSTM layer contains 64 LSTM units and outputs a 64-dimensional feature vector. Then, a Dense layer with 64 neurons follows, performing feature extraction and non-linear transformation. The processed features then flow into the centralized processing module, which consists of two parallel Dense layers and is responsible for separately estimating the state value and action advantage. The state value stream uses 1 neuron to output a scalar V representing the overall quality of the state, while the action advantage stream employs 2 neurons to output the advantage A of each action relative to the average level. Finally, the *Q*-values are computed through the Lambda layer, and a 2-dimensional *Q*-value vector is output for action selection.

The LSTM layer is positioned between the input layer and the fully connected layer to process sequential input states and capture dependencies in the time-series data. In typical reinforcement learning scenarios, the environment is usually assumed to follow a Markov decision process, where the future state is only related to the current state and the action taken. However, the actual environments are often more complex and may not strictly adhere to the Markov property [31]. In such cases, the incorporation of an LSTM layer enhances the model’s ability to retain and integrate historical information, thereby enabling the network to learn from long-term sequential data and improve its capacity for optimizing cumulative rewards over extended periods.

The design of the centralized processing module follows the method proposed in [32]. The centralized processing module consists of two parallel fully connected layers, which decompose the input state into a state value function V and an action advantage function A. The module then performs centralized processing on the action advantage function to calculate the final *Q*-value as follows:

(9) $$Q\left(s_{t},a;\theta ,\alpha ,\beta \right)=V\left(s_{t};\theta ,\beta \right)+(A\left(s_{t},a;\theta ,\alpha \right)-\frac{1}{\left|A\right|}\sum_{a^{\prime }}A(s_{t},a^{\prime };\theta ,\alpha ))$$

where $Q\left(s_{t},a;\theta ,\alpha ,\beta \right)$ denotes the *Q*-value when the input state is $s_{t}$ and the corresponding action is a; $V\left(s_{t};\theta ,\beta \right)$ denotes the state value function V when the input state is $s_{t}$; $A\left(s_{t},a;\theta ,\alpha \right)$ denotes the action advantage function A when the input state is $s_{t}$ and the corresponding action is a; $\left|A\right|$ denotes the number of selectable actions; $\sum_{a^{\prime }}A(s_{t},a^{\prime };\theta ,\alpha )$ denotes the sum of action advantage functions when the input state is $s_{t}$; α and β denote the network parameters of the two fully connected layers, respectively; θ denotes the network parameters of the DDQN main network; and a and $a^{\prime }$ denote action parameters. This structure enables the network to update the state value function V in each training step while simultaneously influencing the *Q*-values of all actions. As a result, the proposed neural network structure can learn the state-value function more frequently and accurately.

### 4.3. UASN MAC Protocol Based on the Proposed DRL Model

Based on the proposed delay-fluctuation-resistant DRL model, we develop a MAC protocol for centralized UASNs named DFR-DLMA (Delay-Fluctuation-Resistant Deep Reinforcement Learning Multiple Access). The centralized UASN consists of a master node and N slave nodes. The master node, typically a surface buoy, performs preliminary processing on the ocean data collected from the slave nodes before forwarding it to an onshore remote base station or monitoring center for in-depth analysis and storage. The slave nodes are underwater sensors responsible for acquiring marine environmental data and transmitting it to the master node via uplink underwater acoustic channels. The slave nodes are categorized into two types: the first type operates using the TDMA protocol, transmitting data packets within specific time slots allocated by the master node. The second type employs the DRL protocol, utilizing idle time slots that are not occupied by TDMA nodes to send data. In the TDMA protocol, both data packets and acknowledgment (ACK) signals are of fixed length. Each time slot is divided into two parts: the first part is used by the slave node to transmit data packets, and the second part is used by the master node to broadcast ACK signals. A guard interval, which may span one or multiple time slots, is set to exceed the maximum transmission delay in the sensor network to avoid interference between packets from different nodes during propagation. The DFR-DLMA protocol is similar to DR-DLMA [11] but incorporates the proposed DRL model and involves the following steps:

1. Network Initialization: The master node first broadcasts a beacon packet. Each slave node calculates the number of propagation time slots between itself and the master node based on the received packet.
2. DFR-DLMA Node Initialization: Initialize the DDQN algorithm by defining its key components (agent, action, state, and reward). Also, initialize the experience replay buffer for these nodes.
3. Action Selection and Coordination: Each DFR-DLMA node inputs the initial state into the DDQN main network to obtain *Q*-values for all possible actions. Using the ε-greedy strategy, the node selects an action, either “Transmit” or “Wait”. One DFR-DLMA node is designated as the gateway, which is connected to all other DFR-DLMA nodes via a control channel for coordination. In each time slot, the gateway determines whether DFR-DLMA nodes should transmit. If the action chosen is “Transmit”, the gateway selects one node via polling to send a data packet to the master node. Otherwise, all nodes remain in the waiting state.
4. Acknowledgement Handling: After the master node successfully receives a data packet, it broadcasts an ACK signal to confirm reception. If no ACK is broadcasted, it indicates that a collision occurred during the transmission in Step 3.
5. Experience Processing and Training: After transmitting, each DFR-DLMA node observes the presence or absence of an ACK signal, computes the corresponding reward, and transitions to the next state. These sequential experiences are stored in the replay buffer and are used to train the DDQN network. The *Q*-values are continually updated to optimize the transmission strategy. ACK failures are handled differently based on their nature: persistent failures are treated as communication interruptions, while occasional failures are corrected through subsequent algorithm training.
6. Strategy Execution: Each DFR-DLMA node transmits data packets to the master node according to the optimal transmission strategy obtained in Step 5.
7. Convergence Assurance: Execute the protection training mechanism [11] to drive the average reward to a steady state, thereby maximizing sensor network throughput.

## 5. Simulations and Results

### 5.1. Simulation Setup

In the simulations, we consider the same centralized UASN model with the same configuration as in [11]. The master node and slave nodes are fully connected and communicate in half-duplex mode. Each slave node operates its own MAC protocol and transmits data packets to the master node in a time-slotted manner. Nodes are randomly distributed within a 10 km×10 km×1 km monitoring area. The underwater acoustic speed is fixed at 1500 m/s and the time slot duration is set to 200 ms. The data packet size is 256 bits and the ACK packet size is 16 bits, with a data transmission rate of 1600 bit/s.

To evaluate the robustness of the proposed protocol against delay fluctuations, we introduce variations into the propagation delay by modeling it as a Gaussian-distributed random variable [1,33,34]. The mean of the delay is set to the number of time slots required for information propagation between any two nodes in the absence of delay fluctuations. Considering that longer propagation delays between nodes are more susceptible to environmental interference and thus exhibit greater fluctuations, the variance of the delay is set as a percentage of the mean delay in the simulations.

The proposed DRL network is implemented using the Python 3.9.12 programming language and the Keras [35] deep learning framework. We referred to the parameter ranges commonly used in DR-DLMA [11] and DRL related works. Building on this, we conducted systematic parameter sensitivity analysis and ablation experiments on the validation set for the proposed DRL network, ultimately determining the parameters. The parameter settings of the proposed DRL network are shown in Table 1.

### 5.2. Performance Evaluation Metrics

To comprehensively evaluate the performance of the proposed DFR-DLMA protocol, the following three key metrics are employed: average training time, short-term average network throughput and normalized throughput [36,37].

Average training time of the algorithm refers to the average time required for the algorithm to converge, measured in time slots. In this paper, an algorithm is considered to have converged when its short-term average network throughput reaches 0.996. The value 0.996 is used exclusively for result analysis and performance evaluation, while a dynamic convergence detection mechanism is employed during model training. The mechanism employs a 1600-slot window to monitor the average reward. It maintains the most recent 1600 time slots of data and computes the corresponding average reward. Convergence is considered achieved when the fluctuation of the average reward within the window remains below 5%. This method overcomes the limitations of fixed thresholds and adapts to varying load conditions.

Short-term average network throughput is a normalized value defined as the ratio of the average amount of data correctly received by the master node over the past $N_{r}$ time slots to the transmission rate. It is calculated as follows:

(10) $$\frac{bits\;per\;packet\times number\;of\;packets\;correctly\;received\;in\;N_{r}\;time\;slots}{transmission\;rate\times N_{r}\times duration\;per\;time\;slot}$$

Normalized throughput is defined as the ratio of the throughput to the transmission rate, calculated as follows:

(11) $$\frac{bits\;per\;packet\times number\;of\;packets\;correctly\;received}{transmission\;rate\times simulation\;time}$$

### 5.3. Comparison with DRL-Based Protocols

Compared to DR-DLMA, the main improvements of the proposed method include integrating delay fluctuation characteristics into the DRL framework and replacing the DQN used in DR-DLMA with an optimized DDQN. To evaluate the effectiveness of these enhancements, simulations were conducted comparing the proposed DFR-DLMA against both the original DR-DLMA and a variant (denoted as DDQN-DLMA) that employs DDQN but does not incorporate delay fluctuation features. The underwater propagation delays for the three protocols follow a Gaussian distribution, with the standard deviations set to 5%, 10%, 20%, and 30% of the mean value, respectively. All three protocols are based on DRL, hence they achieve similar performance upon convergence. However, differences exist in their convergence speed and stability. In the simulations, the average training time and the short-term average throughput are used to evaluate the convergence speed and stability of the algorithms. To simulate the application scenario where DRL-based protocols capture idle time slots, the slave nodes in the underwater acoustic network are divided into two categories in the experiment: DRL nodes and TDMA nodes, with a ratio set to 2:3. The DRL nodes operate using one of the three compared protocols. Experimental results are obtained by averaging over 200 independent simulation runs.

#### 5.3.1. Convergence Speed

**Figure 2:**
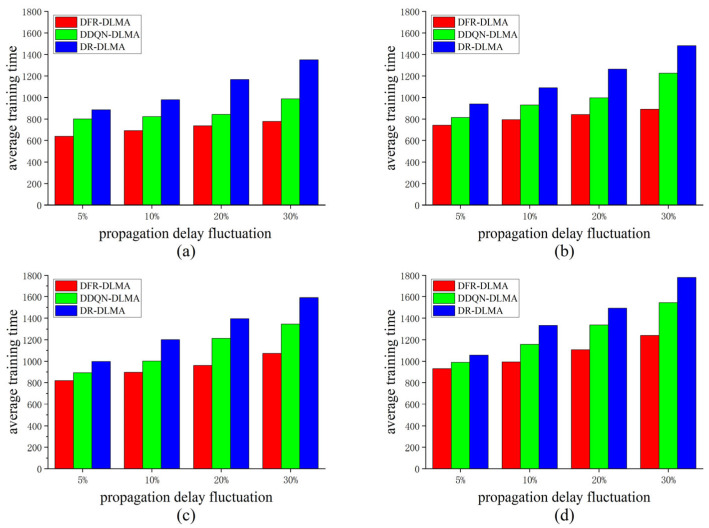
Average training times of the three protocols under different propagation delay fluctuations and different numbers of nodes. (**a**) *N* = 5; (**b**) *N* = 10; (**c**) *N* = 15; (**d**) *N* = 20.

This paper employs the average training time of the algorithms to evaluate the convergence speed, considering the algorithm converged when the throughput reaches 0.996. The convergence speeds of the three protocols are compared under different propagation delay fluctuations and different numbers of nodes. The experimental results are shown in Figure 2.

As shown in Figure 2, DFR-DLMA requires the fewest training times under the same number of nodes and same propagation delay fluctuation. In terms of training speed, it achieves an average improvement of 29.3% and 15.5% over DR-DLMA and DDQN-DLMA, respectively, demonstrating superior learning efficiency and convergence performance. When the number of nodes is fixed, the training times of DFR-DLMA do not increase significantly as the propagation delay fluctuation grows. Similarly, under a fixed delay fluctuation, although all protocols require more training times as the number of nodes increases, the rate of increase for DFR-DLMA is notably lower than that of the other two protocols. These results suggest that due to the incorporation of the propagation delay fluctuation feature, the proposed protocol exhibits enhanced robustness and better adaptability to dynamic network conditions. Additionally, the faster convergence speed of DDQN-DLMA compared to DR-DLMA further demonstrates that the proposed network architecture achieves higher learning efficiency than the conventional DQN used in DR-DLMA.

#### 5.3.2. Stability

**Figure 3:**
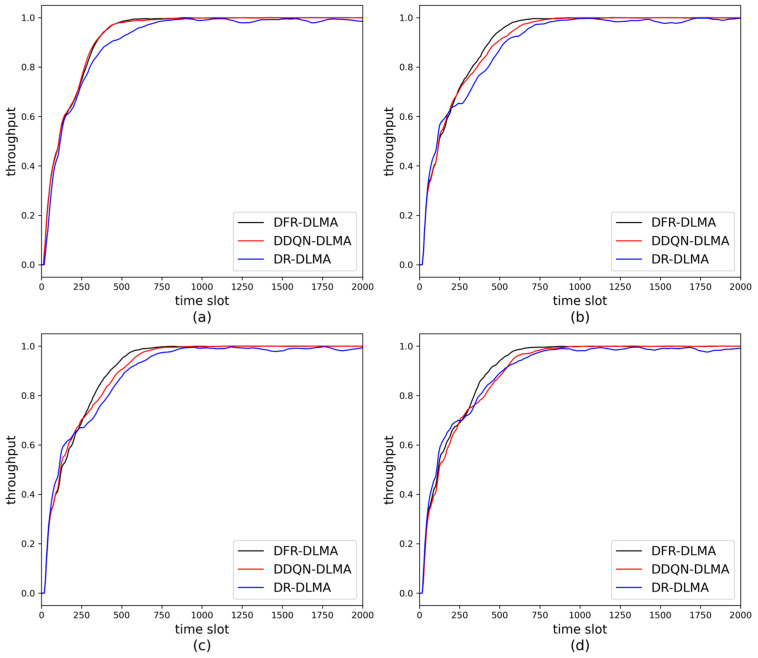
Short-term average network throughput of the three protocols under different propagation delay fluctuations. (**a**) 5%; (**b**) 10%; (**c**) 20%; (**d**) 30%.

The short-term average network throughput is employed to evaluate the stability of different algorithms after convergence. The number of nodes in the experiment is set to 5. The experimental results are shown in Figure 3. As shown in the figure, DFR-DLMA converges faster than the other two protocols. The short-term average network throughputs of all three protocols approach 1 after convergence, and the throughput of DFR-DLMA at convergence is slightly higher than that of the other two protocols. As the propagation delay fluctuation increases, the convergence speed of all three protocols decreases as the fluctuation increases. This is because the heightened delay fluctuation leads to greater environmental uncertainty, requiring the algorithms more time for exploration and learning. Moreover, both DFR-DLMA and DDQN-DLMA maintain relatively stable throughput levels after convergence. In contrast, the throughput of DR-DLMA still fluctuates significantly after convergence. This difference can be attributed to the use of DDQN in the first two protocols, which mitigates *Q*-value overestimation bias and thus enhances stability.

#### 5.3.3. Ablation Experiments

To more clearly isolate the contribution of each component to the model performance, we conducted ablation experiments on individual components. The ablation experiments were performed under the conditions of 5 nodes and 5% delay fluctuation, using the average training time as the metric. All experiments maintained the same experimental environments and parameter settings to ensure validity and comparability of the results. Experimental results are presented in Table 2, where a “√” indicates that the corresponding component was included in the experimental configuration.

Through a comprehensive analysis of the ablation experiment results, we can draw the following conclusions. We adopted the original DR-DLMA algorithm as the baseline (Experiment 1), with an average training time of 885. Experiment 3 introduced delay fluctuation ΔD on DR-DLMA, which increased the average training time by 9.9%. It demonstrates that the DR-DLMA protocol has limited resistance to delay fluctuations. From the single-component experiments (Experiments 2, 4, and 5), it can be observed that DDQN, LSTM, and the centralized processing module (CPM) contributed positively to resisting delay fluctuation. Experiment 6 employed DDQN under delay fluctuation and reduced the average training time by 12.4%, indicating that DDQN is effective in resisting delay fluctuation. Experiments 7 and 8 combined DDQN with LSTM and the centralized processing module, respectively, leading to reductions in average training time of 10.7% and 11.6%. Experiment 9 implemented the complete DFR-DLMA model proposed in this paper, achieving the lowest average training time, thereby validating the effectiveness of the full model in resisting delay fluctuation.

### 5.4. Comparison with Conventional Protocols

To evaluate the performance of the proposed protocol, the normalized throughput of DFR-DLMA was compared with that of DR-DLMA [11], TDMA [38], and DOTS [39] under different network loads and different numbers of nodes. To more accurately assess the maximum capability of each protocol, all slave nodes in the network employed the same MAC protocol to avoid the performance evaluation being affected by a hybrid network environment.

#### 5.4.1. Normalized Throughput Under Different Network Loads

**Figure 4:**
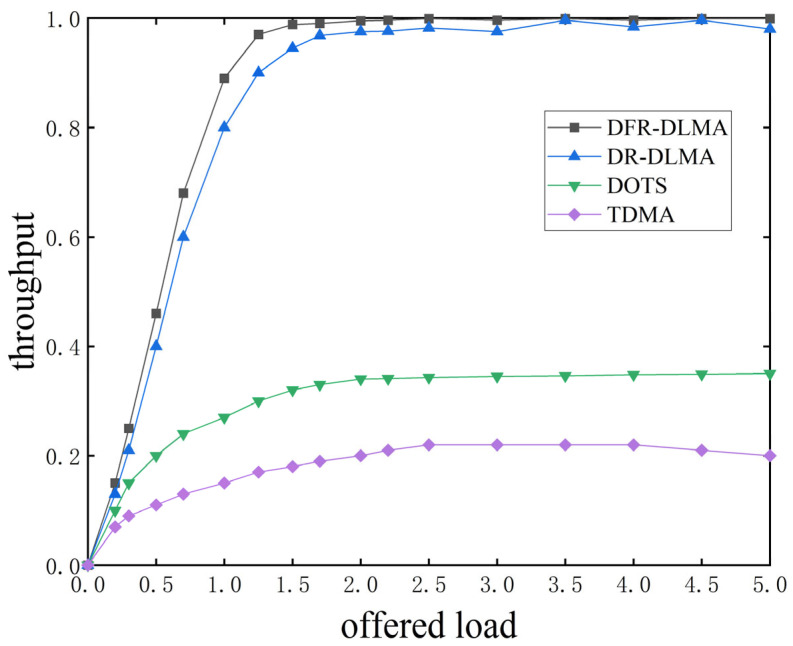
Normalized throughput of the four protocols under different network loads.

Figure 4 presents the normalized throughput of the four protocols across various network loads. The experiment was conducted with 5 nodes and propagation delay fluctuation was set to 5%. The results were averaged over 200 simulation runs.

As shown in Figure 4, the throughput of each protocol increases continuously with rising network load until reaching saturation. After stabilization, the throughput of the DFR-DLMA protocol is 355% and 186% higher than that of the traditional TDMA and DOTS protocols, respectively. The TDMA protocol rigidly allocates channel time to each node, leading to wasted channel resources when nodes have no data to transmit. This inflexibility prevents full utilization of all available time slots, significantly limiting overall network throughput. The DOTS protocol requires notifying other nodes of the transmission schedule via control packets (such as RTS and CTS) before each transmission. This process generates a large amount of overhead, thereby reducing the overall network throughput. Furthermore, the normalized throughput of the DFR-DLMA protocol is slightly higher than that of the DR-DLMA protocol. This improvement can be attributed to the incorporation of delay fluctuation characteristics and the improvement in the DFR-DLMA network architecture.

#### 5.4.2. Normalized Throughput Under Different Numbers of Nodes

**Figure 5:**
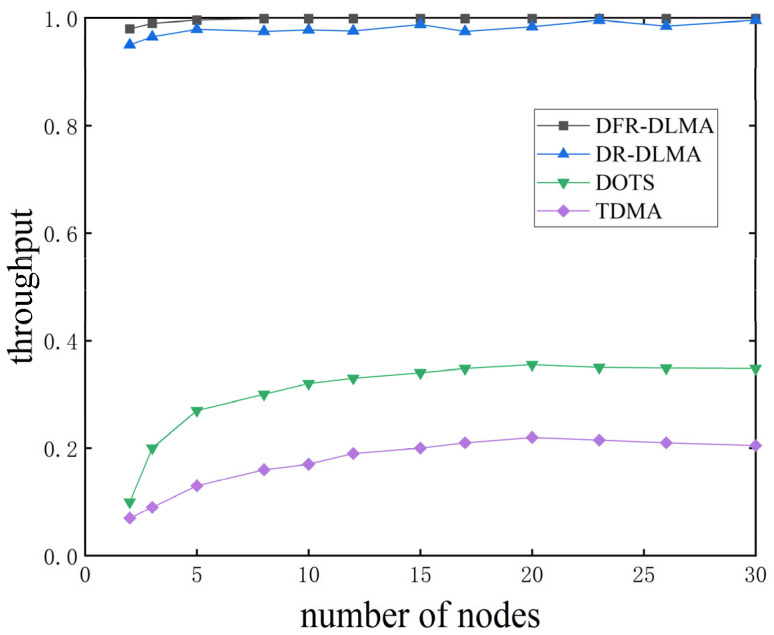
Normalized throughput of the four protocols under different numbers of nodes.

Figure 5 shows the normalized throughput of the four protocols under different numbers of nodes, with the network load fixed at 1.5. The normalized throughput of DFR-DLMA remains consistently high between 0.98 and 1.0 across all tested network scales. Although the normalized throughput of DFR-DLMA is only slightly higher than that of DR-DLMA, it demonstrates advantages in adapting to networks of different sizes. Even as the number of nodes increases, DFR-DLMA is able to maintain high throughput performance. In contrast, the throughput of traditional TDMA and DOTS protocols ceases to increase and stabilizes at a relatively low level once the network size reaches a certain threshold. When the number of nodes exceeds 20, the throughput of TDMA stabilizes at approximately 0.21 and DOTS saturates around 0.35.

### 5.5. Computational Complexity

The computational complexity metric is the total number of floating point operations (FLOPs) during algorithm execution [40]. To provide an intuitive computational complexity benchmark between the proposed method (DFR-DLMA) and the baseline method (DR-DLMA), both the DFR-DLMA and DR-DLMA were executed for 200 runs on an Ubuntu platform (CPU: Intel Core i9-9900K, GPU: NVIDIA GeForce RTX 2080 Ti). The number of nodes is 5, and the delay fluctuation is set to 5%. The FLOPs and average single-run time (AST) are presented in Table 3.

As shown in Table 3, the proposed method requires 57,034 FLOPs, representing an approximately 25% increase over the baseline method. This indicates that DFR-DLMA performs more complex computations. The AST of DFR-DLMA is 13.168 s, significantly longer than the 8.028 s required by DR-DLMA. However, DFR-DLMA demonstrates a lower standard deviation of 0.259 s in single-run time, compared to 0.420 s for DR-DLMA. Correspondingly, the 95% confidence interval (CI) width for DFR-DLMA is 0.073 s, compared to 0.119 s for DR-DLMA, demonstrating that the DFR-DLMA results are more concentrated and exhibit better stability and predictability. In summary, the proposed method achieves enhanced performance stability at the cost of increased computational complexity and extended execution time.

## 6. Conclusions

Conventional DRL-based UASN access schemes often overlook the variability of underwater acoustic link delays and suffer from low learning efficiency. This paper proposes a deep reinforcement learning-based access method for mitigating the impact of propagation delay fluctuations in UASNs. The proposed method explicitly incorporates propagation delay fluctuations into the DRL framework and further employs an optimized neural network architecture to enhance learning efficiency. Simulation results demonstrate that the proposed protocol not only accelerates convergence in environments with variable delays, but also significantly improves the adaptability and stability of the sensor network. Although the proposed method enhances performance, its increased structural complexity leads to higher computational and storage requirements. Future work will focus on reducing energy consumption and balancing performance and efficiency.

## Figures and Tables

**Table 1:** Settings of the proposed DRL network.

Parameter	Value
Number of neurons per layer	64
Activation function	ReLU
State history length M	30
Reward discount factor γ	0.95
Greedy index ε	Decays from 0.1 to 0.001
Experience buffer capacity	500
Number of random samples $N_{E}$	32
optimizer	Adam
Learning rate α	0.01
Target network update frequency F	200
Smoothing window size $N_{r}$	1600
Threshold of reward change	5%

**Table 2:** Ablation experiment results.

	DDQN	∆D	LSTM	CPM	Average Training Time	Difference
1					885	Baseline (DR-DLMA)
2	√				850	−3.9%
3		√			973	+9.9%
4			√		859	−2.9%
5				√	851	−3.8%
6	√	√			775	−12.4%
7	√		√		790	−10.7%
8	√			√	782	−11.6%
9	√	√	√	√	639	−27.8% (DFR-DLMA)

**Table 3:** Computational complexity.

Method	FLOPs	AST (s)	95% CI for AST (s)
DFR-DLMA	57034	13.168 ± 0.259	[13.131, 13.204]
DR-DLMA	45570	8.028 ± 0.420	[7.968, 8.087]

## Data Availability

Data are contained within the article.
